# The start and development of epilepsy surgery in Europe: a historical review

**DOI:** 10.1007/s10143-015-0641-3

**Published:** 2015-05-24

**Authors:** Olaf E. M. G. Schijns, Govert Hoogland, Pieter L. Kubben, Peter J. Koehler

**Affiliations:** Department of Neurosurgery, Maastricht University Medical Centre, PO Box 5800, 6202 AZ Maastricht, The Netherlands; Department of Psychiatry and Neuropsychology, Division Cellular Neuroscience, University of Maastricht, Maastricht, The Netherlands; Academic Centre for Epileptology (ACE), Maastricht University Medical Centre, Maastricht, The Netherlands; Department of Neurology, Atrium Medical Centre, Heerlen, The Netherlands

**Keywords:** History, Epilepsy surgery, Europe, SEEG, Electrode implantation

## Abstract

Epilepsy has not always been considered a brain disease, but was believed to be a demonic possession in the past. Therefore, trepanation was done not only for medical but also for religious or spiritual reasons, originating in the Neolithic period (3000 BC). The earliest documentation of trepanation for epilepsy is found in the writings of the Hippocratic Corpus and consisted mainly of just skull surgery. The transition from skull surgery to brain surgery took place in the middle of the nineteenth century when the insight of epilepsy as a cortical disorder of the brain emerged. This led to the start of modern epilepsy surgery. The pioneer countries in which epilepsy surgery was performed in Europe were the UK, Germany, and The Netherlands. Neurosurgical forerunners like Sir Victor Horsley, William Macewen, Fedor Krause, and Otfrid Foerster started with “modern” epilepsy surgery. Initially, epilepsy surgery was mainly done with the purpose to resect traumatic lesions or large surface tumours. In the course of the twentieth century, this changed to highly specialized microscopic navigation-guided surgery to resect lesional and non-lesional epileptogenic cortex. The development of epilepsy surgery in Southern Europe, which has not been described until now, will be elaborated in this manuscript. To summarize, in this paper, we provide (1) a detailed description of the evolution of European epilepsy surgery with special emphasis on the pioneer countries; (2) novel, never published information about the development of epilepsy surgery in Southern Europe; and (3) we review the historical dichotomy of invasive electrode implantation strategy (Anglo-Saxon surface electrodes versus French-Italian stereoencephalography (SEEG) model).

Appointing the beginning of epilepsy surgery is difficult as epilepsy was not recognized as a chronic neurological disease in the Neolithic period (5500–5000 BC). Due to the supernatural attributions of the origin of the disease, its treatment was not medical but spiritual. From the fourth century BC on, the belief in magical causes and therapies of epilepsy gradually faded into the background. The first descriptions with regard to epilepsy as a disease are found in the Hippocratic Corpus (400 BC), where it was named the “Sacred Disease.” This is the first book, in which epilepsy is localized in the brain [105]. A second observation was that epilepsy is not a divine disease, but an excess of phlegm in the brain which causes the symptoms of an epileptic attack, when it enters the blood vessels.[105] Supporting Hippocrates’ humoral theory, Galen (131–201 AD) combined this with his theory of the four qualities (cold, warm, moist, and dry), which led to his explanation that the “idiopathic” form of epilepsy results from the production of thick, cold humor, phlegm or black bile, in the cerebral ventricles [105]. Evacuation of phlegm would lead to a cure of the epilepsy. The process of diagnosis and treatment of epilepsy stayed obscure in the following centuries till the mid of the nineteenth century [105, 52, 97, 28, 15, 56, 58, 13, 67, 93]. During the seventeenth and eighteenth centuries, epilepsy was one of several areas of debate in the separation process of “nervous disorders” from “mental disorders,” which led to the beginnings of modern neurology in the nineteenth century.

The era of epileptology, strictu sensu, commenced in 1861 (Fig. 1), when John Hughlings Jackson (Yorkshire, UK, 1835–1911), founder of “modern” epileptology, correlated epileptic phenomena to brain dysfunction [104]; in the following years, he described (1868) the corpus striatum as the main seat of the convulsions [45]. In 1870, his concept changed into the hypothesis that seizures originated in the cerebral cortex [46, 55]. By studying unilateral motor seizure symptoms, Jackson was able to conclude that the motor cortex was concerned with movements rather than individual muscles [120]. This was confirmed by preclinical research on cortical stimulation in animals by Sir David Ferrier (1843–1928), a Scottish neurologist/psychologist [19, 20]. Initially, epilepsy surgery was based on identification of visible cortical lesions, usually of traumatic origin. The era of epilepsy surgery began (first operation in 1886) with Sir Victor Horsley (UK, 1857–1916) and William Macewen (UK, 1848–1924), founders of British neurosurgery. Horsley localized and removed lesions in three epilepsy patients at London’s National Hospital at Queen Square and collaborated with Hughlings Jackson [102]. At the same time in Germany, Fedor Krause (1857–1937) started to operate on epilepsy patients. Krause collaborated in Berlin with the well-known German neurologist Hermann Oppenheim (1858–1919). Collaboration between neurologists and neurosurgeons became a prerequisite for successful operations. Multidisciplinary epilepsy surgery working groups, existing in most European countries nowadays, did not begin until the 1970s, first in countries like The Netherlands and the UK.

**Fig. 1:**
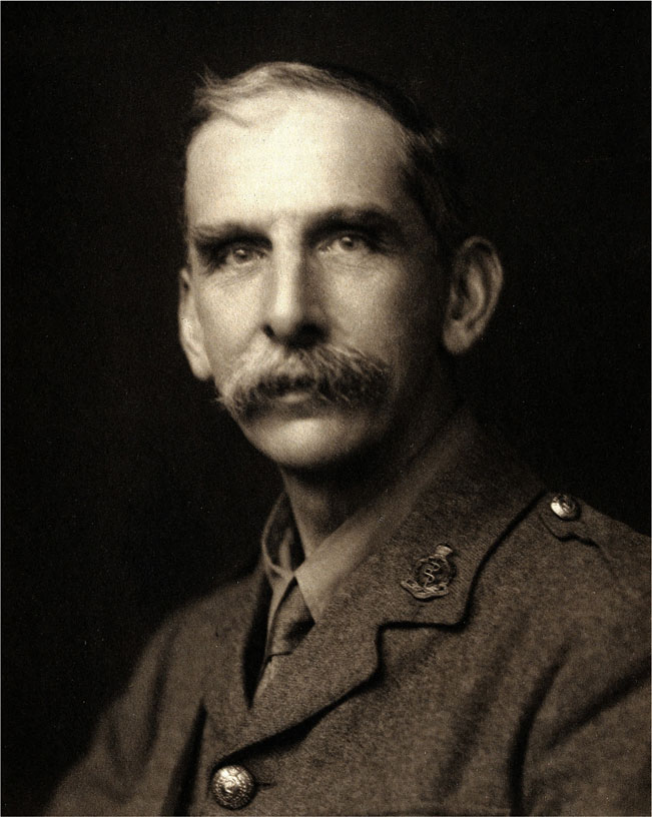
Victor Horsley (1857–1916)

In this paper, we review, from a pioneer’s point of view, the evolution (nineteenth to twentieth century) of modern epilepsy surgery in Europe. The development of epilepsy surgery in Portugal, Spain, and Greece has to our knowledge not been described before.

## Methods

We used MEDLINE/PubMed as a primary data source. Search strategy comprised terms including epilepsy surgery, Europe, European country names, MRI and epilepsy, cortical stimulation, and grid/depth electrodes. Furthermore, a selection of books was consulted, including Temkin’s *The falling sickness*, Wyllie’s *Treatment of Epilepsy* [118], Lüders’ (ed.) *Epilepsy surgery* [67], and *Epilepsy through the ages* [1]. Besides these literature sources, various colleagues in Southern Europe have been asked to provide information on the topic of epilepsy surgery in their respective countries (see Acknowledgments). We chose to start in the Hippocratic period, when naturally observable phenomena instead of magical explanations were held responsible for diseases and more somatic causes were searched for as explanation of the observed phenomena. We confined ourselves to the topic of epilepsy surgery in Western, Southern, and Northern Europe. Especially the development in the pioneer countries, Germany, UK, and The Netherlands, where modern epilepsy surgery had its roots, was described and emphasis was given to the historical development and the actual situation in Portugal, Spain, and Greece.

## Results

### The start of epilepsy surgery in Europe in three pioneering countries: UK, Germany, and The Netherlands

#### UK

##### John Hughlings Jackson, Sir Victor Horsley and William Macewen

Hughlings Jackson is considered “the father” of epileptology. He worked as a neurologist in the National Hospital for the Paralysed and Epileptic, London (1862) together with Brown-Séquard (1817–1894), who was considered an expert in epilepsy at the time. The analysis of epileptic symptoms by Hughlings Jackson was strongly influenced by the evolution and dissolution theory of Von Baer (1792–1876) and Spencer (1820–1903) [47, 98]. This led, in the period 1862–1868, to his concept of a hierarchical organization of the central nervous system with three centers from low to high complexity: (a) spinal cord and brain stem, (b) cortex, and (c) the prefrontal gyri [119], where epileptic seizures could originate: spinal fits (spinal cord) [54], epileptiform fits (cortex), and epileptic fits (prefrontal gyri) [106]. In the period 1868–1870, he emphasized the relation between epileptic seizures and abnormal cortical discharges already before publication (1870) of the cortical stimulation experiments of Fritsch (1838–1891) and Hitzig (1838–1907).[106] Jackson clarified that the mechanism underlying focal and generalized epileptic seizures was similar. The era of epilepsy surgery started in 1886 with pioneer Sir Victor Horsley (Fig. 1). In that year, he operated a patient with a depressed skull fracture and Jacksonian epilepsy. Following trepanation, he removed scar tissue from the cortex bordering the superior frontal sulcus, which led to disappearance of the seizures [40]. Many of his patients suffered from post-traumatic epilepsy, and because of the scarcity of antiepileptic drugs, surgery often was the only remedy. In 1888, Horsley suggested that in the Neolithic period, the reason for placing burr holes above the central area may have related to the epilepsy syndrome of the patients. He came to this suggestion because it was accepted at his time that Jacksonian epilepsy had its origin in the motor cortex [41].

The second British neurosurgical pioneer with interest in epilepsy surgery was William Macewen, since 1892 professor of surgery in Glasgow and pioneer with the introduction of the endotracheal intubation [68, 70]. His first neurosurgical, post-mortem, operation was performed in 1876 in a boy, who died because of a brain abscess. Already prior to Horsley, between 1876 and 1884, he has operated on brain tumour patients using cortical function localization as a method to reach the lesion [69]. He was the first to combine technologies of anesthesia, antisepsis, asepsis, and cortical localization. At that time, Sir William Osler (1849–1919) was a strong supporter to develop neurosurgery as a separate specialty and pleaded (1907) for “medico-chirurgical neurologists who were trained in both neurology and surgery” [18]. During subsequent years, until the early 1950s, neurosurgery evolved into a modern specialty, in particular due to increasing knowledge of intracranial pressure and experiences with brain injuries, but no special innovations with respect to epilepsy surgery were observed. In 1949, Murray Falconer (1910–1977), of New Zealand and trained in England (1938–1943, under Sir Hugh Cairns), was invited to become a consultant neurosurgeon to Guy’s Hospital and the Bethlem Royal and Maudsley hospitals. In 1950, after visiting Penfield in the Montreal Neurological Institute, he started a neurosurgical department at Maudsley Hospital, London. Epilepsy surgery became a main topic in his department, and temporal lobe surgery with acute electrocorticography was the most frequent surgical intervention [17]. He and Professor Meyer developed a technique to resect the complete temporal pole including the mesial structures [94]. Due to this surgical strategy, mesial temporal sclerosis as frequent pathological entity was recognized by his group and focal cortical dysplasia in children with temporal lobe epilepsy was described in cooperation with David Taylor, a child psychiatrist, Manchester, UK [103]. In 1975, he was succeeded by Charles E. Polkey.

After the advent of the MRI scan, it became clear that many chronic epilepsy patients had a structural lesion that could be held responsible for the intractable seizures and was amenable for surgery. It was recognized too that in other patients, still, no structural intracranial abnormality could be diagnosed. With the development of new MRI sequences, small malformations of cortical development (MCD), like, e.g., focal cortical dysplasias (FCD), could be visualized. To answer the question if these lesions were responsible for the seizures, multidisciplinary working groups for epilepsy surgery have been set up throughout Europe. Apart from London, epilepsy surgery programs started in Oxford, Liverpool, Birmingham, and Newcastle. These working groups consisted of epileptologists, neurosurgeons, neuroradiologists, and neuropsychologists with a shared responsibility to diagnose the epilepsy, to clarify the relation between the epilepsy and a radiologically diagnosed lesion, to estimate the epileptogenic zone, and eventually to resect the epileptogenic lesion or cortical area. An interesting observation is that from the start of the comprehensive epilepsy surgery workup, the Anglo-Saxon epilepsy program emphasized the visualization of structural lesions and the correlation of the lesion to the epileptogenic focus, whereas the French-Italian program rather focused on neurophysiological processes and the existence of epileptogenic networks. The cause of this latter approach was the interest of neurosurgeon Talairach (1911–2007) in stereotactic defining cerebral structures together with the interest of neurologist Bancaud (1921–1993) to localize EEG discharges in deep brain structures (see “The evolution of epilepsy surgery and especially the stereo-EEG in France”). Recently, a 1-year survey has been published regarding all epilepsy surgery procedures in the UK in the period 2010–2011. The main conclusion of that report was that there was a decline of temporal and extra-temporal resective procedures and an increase in vagal nerve stimulator implantations [78].

#### Germany

**Fig. 2:**
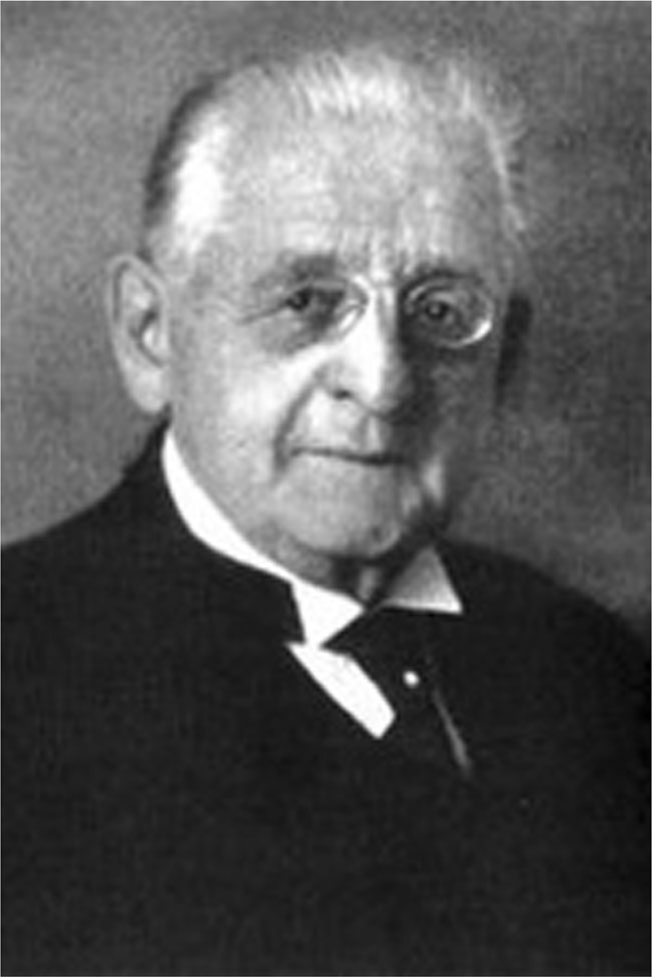
Fedor Krause (1857–1937)

Wilhelm Griesinger (1817–1868), one of the founding fathers of scientific German psychiatry (that comprised neurology as well), argued (1845) that mental disorders had an origin in the brain [30]. After his nomination as a chair of the department of (neuro)psychiatry at the University of Berlin-Charité in 1865, he started an epilepsy service (1866). He proposed the term psychomotor epilepsy (as a synonym for what is presently known as complex partial seizures beginning in the temporal lobe) [65] and tried to correlate epileptic semiology to the pathologic lesion. As director of the Allenberg psychiatry clinic, Sommer (1852–1900) described the typical sclerosis in the Ammon’s horn, also called Sommer’s sector [96]. This hippocampal sclerosis later appeared to be the most frequently encountered pathology in patients with intractable temporal lobe epilepsy [48]. In Berlin, 1870, the neuropsychiatrist Hitzig and anatomist Fritsch collaborated on the electric localization of the motor areas of the brain [34]. They stimulated the motor cortex of awake dogs and were the first to observe that stimulating different areas led to involuntary muscle contractions in different parts of the dog’s body. This was an important step in proving cerebral localization. With this technique, Hitzig and Fritsch inspired other physiologists, including Ferrier (1843–1928) who cooperated with Hughlings Jackson, and may be considered precursors of Penfield and Jasper (Fritsch and Hitzig, 1870). Epilepsy surgery in Germany started with Fedor Krause (1857–1937) (Fig. 2). He devoted himself to the field of neurosurgery, in particular epilepsy surgery, and started electrostimulation of the human motor cortex to design what later became known as the homunculus [67]. He operated over 400 epilepsy patients and published a book on epilepsy in 1931 [66]. He proposed that surgical resection of the ictal onset zone is the most important intervention in order to achieve a successful outcome. A contemporary in the field of epilepsy surgery was Otfrid Foerster, a neurologist and neurosurgeon (1873–1941). He studied medicine in Freiburg, Kiel, and Breslau; became a pupil of Carl Wernicke in Breslau; and studied with Joseph Babinski, Jules Dejerine, and Pierre Marie in Paris. By cooperating with Wernicke, well known for his localization of sensory aphasia in the temporal lobe (1873), Foerster became enthusiastic for localization studies. They published an anatomical atlas of the brain [112]. In 1911, he became chair of the first neurology department separate from psychiatry in Germany [101]. Practicing neurology and later neurosurgery in Breslau, he was frequently visited by colleagues, including Penfield, who continued the work of Foerster in stimulating and mapping the human cortex. Foerster was a pioneer in the field of awake craniotomies, during which he stimulated the motor cortex of awake patients, most of them were veterans from World War I with chronic epilepsy due to traumatic scarred cortex [90]. In collaboration with Penfield, he published the results on the surgical treatment of traumatic epilepsy [21]. Interpretation of seizure semiology was based on these anatomical studies because EEG and electrocorticography (ECoG) did not exist at that time. In 1924, Berger (1873–1941), neuropsychiatrist in Jena, Germany, was the first to measure the electrical activity of the human brain, although he described the invention and application of the human EEG only in 1929 [10]. Between 1940 and the mid-1980s, epilepsy surgery was done at a low volume basis in Germany because of the consequences of World War II and the difficult post-war circumstances. From the 1980s on, several multidisciplinary epilepsy surgery centers started (Bonn, Bielefeld, Berlin, Erlangen, Freiburg, Munich, Greifswald, Kehl-Kork, and Vogtareuth) with the financial support of the German Federal Ministry of Health.

#### The Netherlands and Belgium

The first epilepsy surgery in The Netherlands took place in 1889, when the surgeon Guldenarm (1852–1905) in collaboration with Winkler (Utrecht, 1855–1941), a neuropsychiatrist, operated on a patient with Jacksonian seizures due to a frontal tumour [116]. They continued operating, mainly brain tumour patients, assisted by a new system for defining the exact localization of the cerebral gyri and sulci, the triangulation technique invented by Winkler [53, 117]. In 1896, Winkler moved to Amsterdam where he became professor of neurology and psychiatry and continued epilepsy surgery with the surgeons Korteweg (1851–1930) and Rotgans (1859–1948). In 1897, the first cortical electrostimulation and intraoperative seizure monitoring were performed in The Netherlands, perhaps the first in Europe [108]. Neuropsychiatrist Muskens (1872–1937) started to work in The Hague and Amsterdam after spending some years in the USA and England working, among others, with Victor Horsley to train in epilepsy surgery. In 1902, he founded the “Dutch Society Against Falling Sickness,” and in 1909, he and his Hungarian colleague Donath founded the “International League Against Epilepsy” (ILAE). With the appointment (1923) of Bernard Brouwer as the first professor of (independent) neurology in Amsterdam, a new era with regard to neurosurgery started in The Netherlands. He recognized professional neurosurgery could only be reached by training surgeons specifically in neurosurgery. Following a lecture tour in the USA (1926), he sent Ignaz Oljenick (1888–1981), a resident surgeon, to the USA to train with Cushing [53, 57]. In 1929, Oljenick returned to Amsterdam and started neurosurgery on Brouwer’s department. His pupil, de Vet (1904–2001), was the first neurosurgical resident in The Netherlands; he developed a special interest in epilepsy surgery, resulting in a neurosurgical consultant function at “Meer en Bosch” epilepsy center (Heemstede, The Netherlands). From 1938 to 1942, about 40 epilepsy operations were performed [16]. After the war, neurosurgical procedures continued in Heemstede in 1949. Acute electrocorticography was introduced by Otto Magnus, who had completed a fellowship at the clinic of Jasper (1906–1999) and Penfield (1891–1976) in Montreal. In the period from 1936 up to 1969, when de Vet retired, 213 patients underwent surgery for epilepsy. After his retirement, a new Dutch collaborative epilepsy surgery program was started in 1973. In 1976, the first video-EEG monitoring unit was created in “Meer and Bosch” [12]. Using implanted strip and depth electrodes prior to an eventual resective procedure, intracranial epileptic phenomena were monitored here for the first time in The Netherlands. Surgical procedures (775 between 1973 and 2005) were carried out at the University Hospital of Utrecht. In 1997 and in 2002, the University Hospital Maastricht and Free University Amsterdam, respectively, received permission from the Ministry of Health for the development of an epilepsy surgery program. In 2008, as an addition to the grid, strip, and intrahippocampal depth electrodes (Anglo-Saxon model), the SEEG as an invasive diagnostic procedure (French-Italian model) was introduced by the epilepsy surgery working group of Kempenhaeghe epilepsy center (Heeze) and the neurosurgical department of the University Hospital Maastricht. Nowadays, three Dutch epilepsy surgery centers (Amsterdam, Maastricht, Utrecht) exist. Every center has its own epilepsy surgery working group next to a national working group in which members of the local groups are participating and discussing their patients. The number of operations in The Netherlands will approximately be 150 patients each year, excluding the vagus nerve stimulation (VNS) implantations. As reported in the UK, the number of VNS implantations did the last years also rise in The Netherlands since introduction of this device.

In Belgium, the interest in epilepsy and its surgical treatment started some years before World War I, but surgery started in various university hospitals after the war, including Brussels (first) and later Leuven and Liège. The neurosurgeons received part of their neurosurgical training in the USA. Dedicated epilepsy care started in four centers (Brussels, Leuven, Ghent, and Liège) in 1955. The number of surgical procedures was small, and many patients were treated in The Netherlands. In the beginning of the 1980s, the first series of epilepsy surgery, combined with invasive diagnostic procedures like electrode implantation, started in Hôpital Erasme, Brussels [109]. In 1990, the first multidisciplinary epilepsy center started at the University of Ghent followed at approximately the same time by the University of Liège and at the end of the 1990s by the University of Leuven. Today, four (Ghent, Leuven, and two centers in Brussels) epilepsy reference centers exist in Belgium.

### The evolution of epilepsy surgery and especially the stereo-EEG in France

**Fig. 3:**
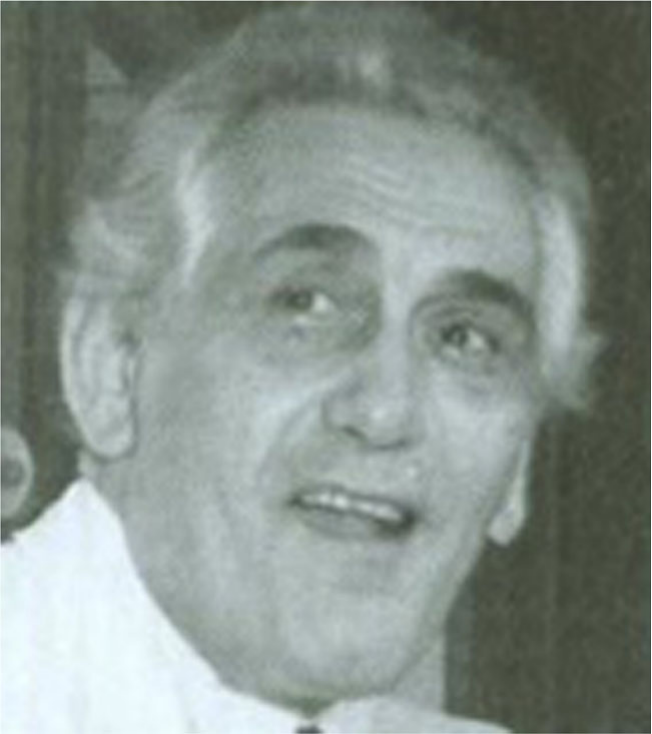
Jean Talairach (1911–2007)

**Fig. 4:**
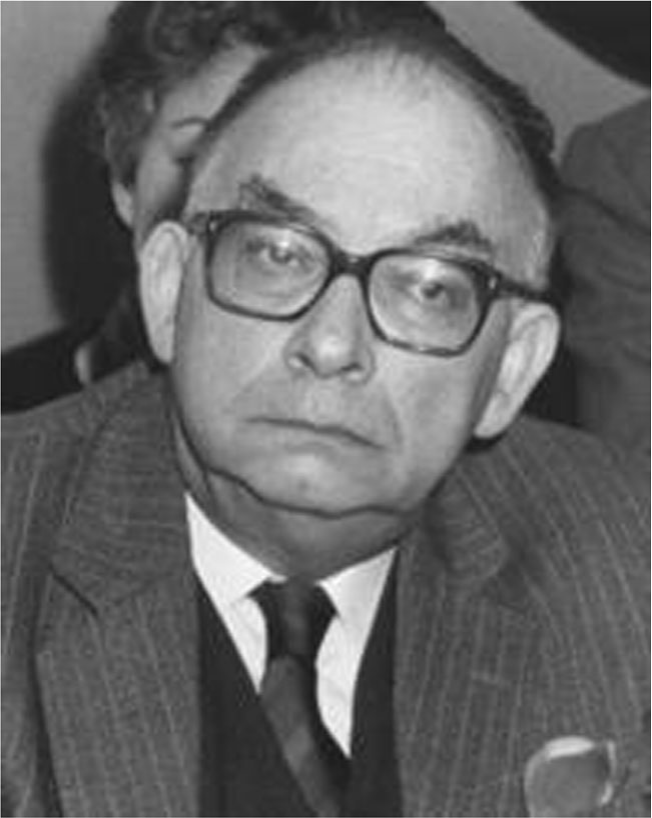
Jean Bancaud (1921–1993)

Epilepsy surgery started in the beginning of the 1950s, when neurosurgeons Mazars and Guillaume, encouraged by Henri Hécaen (1912–1983), a neurologist/neuropsychologist, who did a fellowship in the Montreal Neurological Institute (MNI), started an epilepsy surgery service at St. Anne Hospital in Paris. They performed operations [31–33], assisted by acute electrocorticography. They were the first to describe epileptic seizures probably originating in the insula and the technique to operate on this “fifth lobe” of the brain (Guillaume MMJ). In the same hospital, at the same time, another neurosurgeon, Talairach (1911–2007) (Fig. 3), did an important work on stereotactical techniques, publishing the first stereotactic atlas of deep brain nuclei in 1957 (Talairach). In the early 1950s, he started a collaboration with neurologist Bancaud (1921–1993) (Fig. 4), who worked at La Salpêtrière Hospital in Paris, but then joined the St. Anne group. Bancaud had been influenced by the cortical mapping work of Penfield. In those days, Bancaud realized that the electrophysiological methods to assess the localization of seizure onset were not sufficient. In collaboration with Talairach, using his stereotactical device, they pioneered with the stereotactical implantation of depth electrodes [3, 8, 99], named “stereoencephalography” (SEEG) in 1962 [8]. With this new technique, the spatiotemporal electrical distribution could be detected and correlated to seizure semiology. The SEEG, as a method for detection of the seizure onset zone and the epileptogenic network [51], formed the basis for the preoperative workup of epilepsy patients in France. In the 1960s, the SEEG flourished and the two pioneers described the role of frontal and temporal brain structures in the development of intractable epilepsies [4–6] and used these electrodes also to detect “silent” brain zones, as an indication for a lesion, which at that time could not yet be visualized by CT or MRI scan.

In 1974, the St. Anne group presented a survey of more than 200 operated epilepsy patients (1957–1973) [100]. The SEEG technique was improved, and chronic extra-operative seizure detection became available. After the registration of seizures, stimulation of brain areas also belonged to the possibilities, which enabled them to describe certain automatisms [7]. By implantation in different brain lobes, they were able to describe the multilobar origin of seizures [75], which could alter resection strategy. Several SEEG-based clinical studies have been published; among others on the significance of the insular cortex in the generation and spread of seizures [43, 44, 84] and the description of different types of mesio-temporal lobe seizures [9, 14]. Today, 13 epilepsy surgery centers exist in France (Lille, Paris (4x), Strasbourg, Rennes, Lyon, Grenoble, Bordeaux, Toulouse, Montpellier, and Marseille), and there is a multidisciplinary center for children and adolescents with epilepsy (IDEE) at the University of Lyon. The hallmark of French epilepsy surgery will remain the introduction and elaboration of the SEEG technique.

### The history of epilepsy surgery in the Southern European countries

#### The development of epilepsy surgery in Italy

The practice of a multidisciplinary workup of epilepsy patients towards an operation did not exist in Italy until 1994. In 1994, neurosurgeon Claudio Munari (1943–1999) returned to Italy from Paris after completing his clinical and scientific training in epileptology and epilepsy surgery with Bancaud and Talairach at St. Anne hospital. In Milan, he founded the first comprehensive epilepsy center of Italy, collaborated with the neurophysiologists/neurologists L. Tassi and G. Avanzini, and introduced the SEEG technique which he had learned in France [76, 77]. For many years, the invasive preoperative workup in epilepsy surgery within the epileptological community had been divided into the US-Anglo-Saxon versus the French-Italian approach. The foundation of the Munari Institute, as one of the two centers in Italy solely dedicated to epileptology and epilepsy surgery, gave a new dimension to the treatment of epilepsy patients, and many patients from all over Italy came to Milan for surgery [74]. The second center is located in Pozzilli near Rome (since 1998); these two centers have an operating capacity of approximately 200 patients per year. Other neurosurgical departments in which epilepsy surgery is carried out are Bologna, Rome [88], and Siena.

#### The development of epilepsy surgery in Spain

**Fig. 5:**
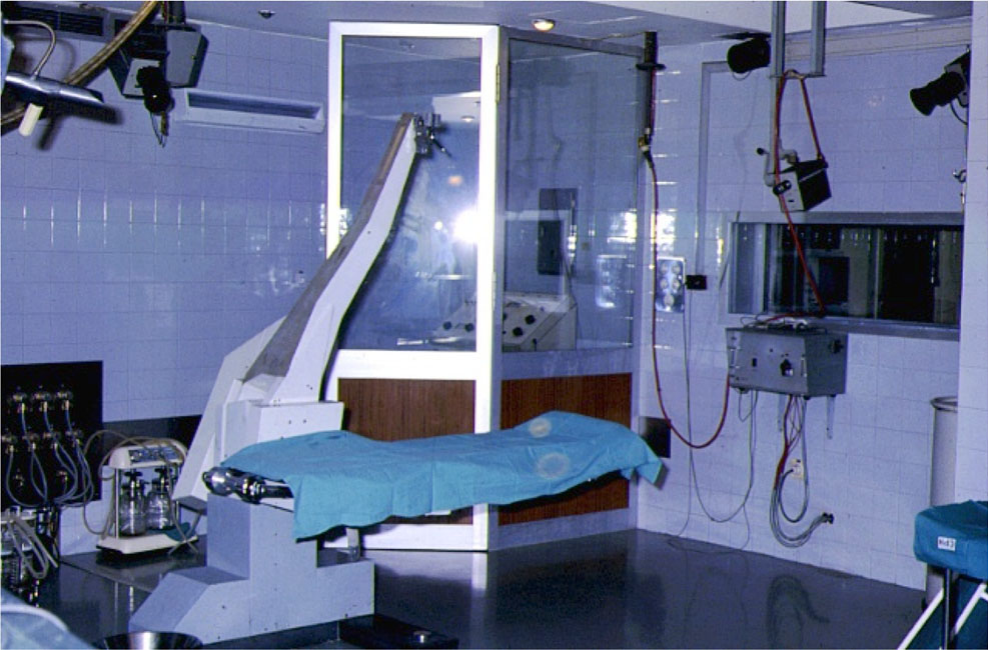
Epilepsy operating room in Clínica Puerta de Hierro, Madrid. Talairach’s surgical table, TV cameras, and x-ray control

In the early 1970s, G. Bravo, head of the neurosurgical department at the Clínica Puerta de Hierro, Madrid, along with neurophysiologist J. Miravet, started with epilepsy surgery in Spain. They designed an operating room for the surgical exploration and treatment of patients with intractable epilepsy, following Talairach and Bancaud’s methodology (Fig. 5).

**Fig. 6:**
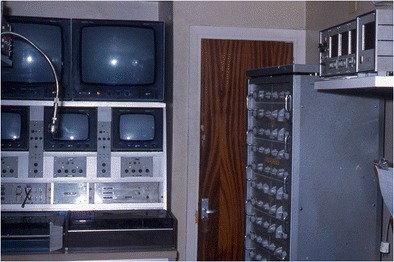
General view of neurophysiological room annexed to the operating room

The outstanding design of the neurophysiology room (Fig. 6), adjacent to the operating room and arranged/equipped by Miravet, should be emphasized. It had four integrated video cameras: three in the operating room and one focused on a digital clock. The clock sends a time signal to one of the 23 channels in the two EEG recorders, enabling to simultaneously record the clinical picture and EEG signal, with an accuracy of almost a tenth of a second. Miravet never published this design, which was probably one of the first simultaneous video-EEG recordings. As described above, the conclusion is that this Spanish design was that of an epilepsy monitoring unit (EMU) “avant la lettre.”

Basically, this operating room design was the first Epilepsy Surgery Unit (ESU) in 1976. In this ESU, M. Manrique performed stereotactic procedures (stereoelectroencephalography), and Bravo carried out cortical resections. In 1978, R.G. Sola was put in charge of the ESU, and from 1980, he performed both types of surgical procedures [23–25]. In 1987, neurosurgeon B. Oliver and neurophysiologist A. Russi set up an ESU at the Clínica Teknon, Barcelona [81]. In 1990, R.G. Sola restarted the ESU in the University Hospital de La Princesa, Madrid. This is probably the Spanish ESU that has developed the highest activity and had the largest number of scientific publications in the last years [73, 83, 86, 87].

Subsequently, other Spanish hospitals started with epilepsy surgery. Hospital Clínic in Barcelona (J. Rumiá), Hospital Virgen de las Nieves in Granada (A. Altuzarra), Hospital Niño Jesús in Madrid (F. Villarejo), Fundación Jiménez Diaz in Madrid (J. Albisua), and Hospital Gregorio Marañon in Madrid (C. Fernández Carballal) have become outstanding ESUs [29].

In 1998, the Ministry of Health carried out a review on epilepsy surgery in Spain, as well as at the international level [29]. This served as a baseline from which the Ministry of Health, at beginning of the year 2000, established a program of Epilepsy Surgery Guidance, officially recognizing 14 centers in Spain.

In December 2010, the Ministry took a similar initiative, creating the CSUR program (Centres, Services and Reference Units), demanding greater levels of activity and quality of care. The Ministry of Health nominated five ESUs in all Spain: University Hospital de La Princesa, Madrid (R.G. Sola), Hospital Clínic of Barcelona (J. Rumiá), Hospital Virgen de las Nieves, Granada (G. Olivares), Hospital de la Fe in Valencia (A. Gutierrez), and Hospital Universitario in Santiago de Compostela (M. Gelabert). Recently, the Hospital de la Paz (Madrid) has been included, and Hospital Niño Jesús (Madrid) is being considered.

Epilepsy surgery is now fully accepted, both by the Spanish society and by the Society of Spanish Neurology. It is estimated that annually at least 200 patients are treated in Spain (40 millions people), with an increasing degree of complexity and good results.

#### The development of epilepsy surgery in Greece

The modern era of epilepsy surgery in Greece started in 1997 in the Evangelismos General Hospital, the largest hospital in Greece. In 2000, following the joint decision of the University of Athens and Evangelismos General Hospital, the Athens University Department of Neurosurgery was located in Evangelismos Hospital. In the following years, a multidisciplinary epilepsy team was organized consisting of neurosurgeons, neurologists, neurophysiologists, neuroradiologists, neuropsychologists, psychiatrists, and epilepsy nurses. Specialized investigations were introduced such as the Wada test, the intraoperative electrocorticography (ECoG), and the intracranial video-EEG monitoring besides the standard presurgical investigations like video EEG, MRI, functional MRI, PET, SPECT, neuronavigation, and neuropsychological and psychiatric assessments,

In 2001, the first standard temporal lobectomies and the first extra-temporal cortical resections were performed [63]. In 2002, the first anterior medial temporal lobectomies and the first selective amygdalohippocampectomies were performed [62].

In 2003, the Epilepsy Surgery Unit was substantially upgraded. The upgraded unit included a fully equipped video-EEG suite, a completely renovated operation theatre, advanced targeting and neuronavigation systems, and upgraded neuroimaging techniques such as functional MRI, MRI spectroscopy, and PET. In 2004, the Athens Epilepsy Surgery Centre was officially inaugurated. Since then, examination of intractable epilepsy cases has become a routine practice. Particularly, in extra-temporal epilepsy cases, immediately after the positioning of intracranial electrodes, the patient undergoes video-EEG monitoring and cortical mapping. A week later, the patient is usually ready for ECoG-guided cortical resections. Multiple subpial transsections are performed in patients, in whom the epileptogenic area is located in eloquent cortical areas [27]. In 2009, the first cortical stimulation procedure was done in a patient with an eloquent cortical epileptogenic zone, and in 2010, the Athens Epilepsy Surgery Centre started performing procedures of deep brain stimulation. Deep brain stimulation (DBS) brain targets included the hippocampus and the anterior thalamic nucleus. The number of epilepsy operations performed between 1997 and 2011 exceeds 200. The strict referral base is a population of approximately 7 million from the area of Athens, South Greece, and the Greek islands, but the actual referral base is much wider including patients from northern Greece and neighboring countries such as Albania, Bulgaria, and Cyprus and also people of Greek descent living in other European countries (personal communication with Prof. S. Gatzonis, 2012).

In St. Luke’s Hospital, a private hospital located in the suburbs of the city of Thessaloniki, in Northern Greece, the Epilepsy Monitoring Unit was established in 2002, initially devoted to diagnostic video-EEG studies with occasionally only non-invasive presurgical evaluations and operations in well-selected lesional, mostly temporal lobe epilepsy, cases. Since 2006 and 2007, the proportion of presurgical evaluations has increased considerably and investigational facilities have been enriched by the addition (in 2009) of fMRI and EEG-fMRI and intracranial electrode monitoring.

Actually, it is a two-bed video-EEG epilepsy monitoring unit performing prolonged video-EEG studies for diagnostic and presurgical evaluation purposes in adults and school-age children. The referral population (city of Thessaloniki and Northern Greece) approaches 2.5 million people. There are also referrals from other areas of Greece and nearby countries (e.g., Albania and Cyprus). In the period 2009–2011, there were 20–25 admissions for presurgical evaluation/year with 10–12 resections/year and 2–4 patients/year who have been implanted with intracranial electrodes. Chronic intracranial electrode monitoring and extra-operative electrical cortical stimulation studies are performed since 2009. Until now, only investigations with combinations of subdural grids and strips have been performed. Future plans include SEEG/depth electrode studies for selected cases. Almost 80 % of the operations were performed for temporal lobe epilepsy by anterior (Spencer type) temporal lobe resection plus amygdalohippocampectomy. The other 20 % of the operations were extra-temporal epilepsy cases with focal cortical resections and occasionally multilobar resections. Acute intraoperative ECoG is utilized in most temporal and extra-temporal cases for confirmation of epileptogenicity and modifications of resections in selected cases [26, 59, 60]. Follow-up information is available on a yearly basis for at least 90 % of the operated patients with 80 % of the patients having an Engel class I outcome, 15 % a class II–III, and 5 % a class IV outcome.

#### The evolution of epilepsy surgery in Portugal

The first Portuguese neurosurgeon to execute the ideas of neurologist Egas Moniz, the Portuguese Nobel Prize winner, was Prof. Almeida Lima (1903–1985), who worked in three different hospitals in Lisbon (Hospital Santa Marta, Hospital Júlio de Matos, and Hospital Santa Maria). He performed the first carotid angiographies and leucotomies in Portugal. He was also the first neurosurgeon, who performed epilepsy surgery in Portugal. His first operation was an anatomical hemispherectomy in a patient with hemiconvulsions and hemiparesis in 1953.

The actual pioneer in epilepsy surgery in Portugal was Dr. Martins Campos, who started in the mid-1970s with temporal lobectomies. He started working in the Hospital Júlio de Matos, Lisbon, and ended his career as chairman of the neurosurgical department (1998–2008) in Hospital Egas Moniz in Lisbon, where he started the epilepsy surgery service. In the beginning of the 1990s, A. Gonçalves Ferreira started epilepsy surgery on a more regular basis in Hospital Santa Maria, Lisbon. At the end of the 1990s, epilepsy surgery, performed by Dr. Fernando Gomes, started in a third center, the University Hospital of Coimbra.

In the north of Portugal, the epilepsy surgery started only very recently, first in Hospital Santo António in Porto, soon followed by Hospital Pedro Hispano in Matosinhos, and finally in a third Northern center, Hospital de São João in Porto.

In summary, six working groups for epilepsy surgery exist in Portugal: (1) Hospital Santa Maria, Lisbon, coordinated by Prof. Jose Pimentel; (2) University Hospital of Coimbra, Dr. Fernando Gomes; (3) Hospital Egas Moniz, Lisbon; (4) Hospital Santo Antonio, Porto, coordinated by Dr. R. Rangel; (5) Hospital Pedro Hispano in Matosinhos; and (6) Hospital Sao Joao, Porto, coordinated by Prof. R. Vaz.

In the two epilepsy surgery centers of Lisbon and in the University Hospital of Coimbra, all known kinds of temporal and extra-temporal operations are performed. The pre-resection invasive diagnostic workup in difficult patients is performed with both subdural grid and strip electrodes, as well as depth electrodes (SEEG). Vagus nerve stimulation (VNS) is concentrated in two centers, namely Hospital Santa Maria in Lisbon and Hospital Santo Antonio in Porto. Deep brain stimulation (DBS) for epilepsy in the anterior nucleus of the thalamus is concentrated in two hospitals, notably Hospital Santa Maria in Lisbon and Hospital Sao Joao in Porto. The epilepsy surgery working groups in Portugal are self-reliant and have relationships with other regional hospitals to collect the epilepsy surgery candidates. Like in many other European countries, there is no centrally coordinating epilepsy surgery working group (personal communication Dr. F. Pinto/Prof. Dr. J. Pimentel).

#### The development of epilepsy surgery in Switzerland and Austria

##### Switzerland

Emil Theodor Kocher (1841–1917), a Swiss general surgeon and Nobel Prize winner, was the first Swiss neurosurgeon who also had a special interest in epilepsy. During his 45 years of professorship and directorship of the university clinic for surgery in Bern, Switzerland, he treated different epilepsy patients, most of them with post-traumatic epilepsy. In 1892, he wrote a manuscript about the treatment of post-traumatic epilepsy in 14 patients in which he described different operative techniques [39]. In their manuscript, Hildebrandt et al. mention Kocher’s remarks about the relationship between intracranial pressure elevation and epileptogenesis. Kocher referred in this subject to the pathophysiological experiments of his colleagues Ito and Berezowsky. He also was one of the first surgeons who emphasized the value of long-term postoperative follow-up of epilepsy patients [39]. Another indication that epilepsy was an important research topic in his department was the visit of Hayazo Ito, a Japanese surgeon and since 1900 the chair of surgery of the Imperial University of Kyoto. Ito developed experimental epilepsy-ICP models and published his findings [39].

The start of neurosurgery in Switzerland as an independent specialty was in 1936 with the opening of a separate neurosurgical department in Zürich chaired in 1939 by Krayenbühl (1902–1985), who received his neurosurgical training with Sir Hugh Cairns in London, UK. Krayenbühl had a great interest in epilepsy surgery, and from the 1950s on, the number of surgical procedures increased [67]. During most of the operations, corticography was applied in the way Wilder Penfield performed his operations. The neurophysiological data were interpreted by neurophysiologist and Nobel prize winner Walter Rudolf Hess who closely collaborated with the neurosurgical department [113]. In 1970, when epilepsy surgery was already routinely performed applying intraoperative neurophysiological monitoring, SEEG was introduced in Switzerland by epileptologist Christoph Bernoulli and neurosurgeon Jean Siegfried. In 1977, Bernoulli described the first case of transmission of Creutzfeldt-Jakob disease by the depth electrodes used in SEEG and this lead to a decrease of the use of the technique in Switzerland [11]. The SEEG technique in combination with the introduction of the operation microscope, as well as the immense spectrum of micro-instruments from 1967 on, undoubtedly led to the different selective resective procedures like the selective amygdalohippocampectomy (sAH), microscopically performed and described by G. Yasargil [114, 119]. Before 1969, most temporal lobe epilepsy patients were treated by a two-thirds standard anterior temporal lobectomy combined with amygdalohippocampectomy. With the introduction of the SEEG in 1970, the strategy changed towards more tailored resections. In 1973, the first selective transsylvian amygdalohippocampectomy, as elaborated by Yasargil [114, 119], was performed based on the idea that the entorhinal cortex is a main area of seizure onset. Nowadays, selective amygdalohippocampectomy is a standard operative procedure in temporal lobe epilepsy all over the world. During the professorship of Yasargil, epilepsy surgery became a common practice in Switzerland, and today, several other centers, including Geneva and Bern, have multidisciplinary epilepsy surgery programs.

##### Austria

In 1904, the first neurosurgical intervention, the treatment of a brain tumor patient, was performed by Anton von Eiselsberg (1860–1939). In cooperation with Egon Ranzi, he operated many patients with brain tumors and epilepsy.

In the period from 1930 to 1939, an independent neurosurgical department arose in Vienna and especially stereotactic procedures for the surgical treatment of epilepsy were performed, including anterior commissurotomy and amygdalotomies. These procedures were carried out by Gangelberger [61], who had been trained by Riechert, a specialist in stereotactic neurosurgery in Freiburg, Germany. Next to these therapeutical procedures, also, invasive electrophysiological diagnostic procedures were performed and published [22, 35]. Following Vienna, a second neurosurgical department opened at the University of Graz in 1950. Diemath introduced stereotactic and functional neurosurgery in 1962, and soon, many epilepsy surgical procedures were performed and experiences published [38]. In 1992, Christoph Baumgartner introduced the first comprehensive program for epilepsy surgery in Austria, and in the same year, radiosurgical treatment for various diagnoses was offered to epilepsy patients [92, 107]. Nowadays, four separate multidisciplinary centers for epilepsy surgery (Vienna, Graz, Linz, and Innsbruck) exist in Austria [67].

### Epilepsy surgery in Scandinavia and Finland

#### Denmark

Epilepsy surgery started here at the University Hospital of Copenhagen in 1960. Dr. Vaernet started with the two-thirds anterior temporal lobectomy as performed in many epilepsy surgery centers all over the world. In collaboration with neurologist Dr. Jensen, he described the results in a paper in 1977 [49]. At the time, one could not yet speak of a comprehensive center for epilepsy surgery. Following a decrease in patient numbers during the 1970s and mid-1980s, there was a revival with the start of a “National Epilepsy Surgery Group” by the neurologist Mogens Dam in 1987. In the beginning, the government restricted the kind of procedures that could be performed because of the cost in relation to the small number of Danish inhabitants. Consequently, many patients who, e.g., needed intracranial diagnostics needed to be transferred to Germany or the USA. Today, there is one comprehensive center for epilepsy surgery for the whole country (“The National Epilepsy Surgery Group” at Rigshospitalet in collaboration with Dianalund Epilepsy Hospital, Copenhagen) in which all diagnostic and resective procedures take place [67].

The principle of centralization of high specialized care is not unique in Denmark but was and is seen also in, e.g., The Netherlands.

#### Norway

In 1949, epilepsy surgery started in Norway as a result of the cooperation between Georg Henriksen, director of the epilepsy center and neurosurgeon Kristian Kristiansen, who was trained by Penfield in Montreal. Having about the same number of inhabitants as Denmark, the epilepsy surgery program has also been centralized since 1976, with one comprehensive center on two locations, notably the National Epilepsy Center in Sandvika and the University Hospital of Oslo where the surgery is performed, approximately 30 operations per year as described by Henriksen [37]. The preoperative workup is similar to many other countries today, from video-EEG until electrode implantation. In case patients need a PET scan and/or a MEG study, patients were referred to, respectively, Sweden and Finland. Patients who have been selected for invasive electrode monitoring received foramen ovale electrodes or subdural grid and strip electrodes [37].

#### Sweden

Before the foundation of modern multidisciplinary centers for epilepsy surgery, Herbert Olivecrona (1891–1980), one of the pioneers of modern neurosurgery, already performed epilepsy surgery in Sweden in the 1950s. In 1935, he became the professor of neurosurgery at the Karolinska Institute, probably the first chair of neurosurgery in Europe. He wrote an interesting article about the future of neurosurgery in which he dedicated a section to epilepsy surgery [80]. Decades later, epilepsy surgery is performed in all six university hospitals in Sweden (Stockholm, Lund, Göteborg, Uppsala, Linköping, and Umeå). In contrast to Denmark and Norway, Sweden has a decentralized program for epilepsy surgery (personal communication; Kristina Källén, Dept. Neurology, Lund, Sweden). All these centers have comprehensive multidisciplinary teams since the 1980s and have reported their results in recent medical literature [72, 82, 95, 89]. The reports were published under the auspices of Swedish National Epilepsy Surgery Register.

#### Finland

Two modern comprehensive epilepsy surgery programs exist here, notably at the University Hospital of Kuopio since 1988 and at the University Hospital of Helsinki. The Kuopio group published their surgery outcome results for the temporal lobe cohort [42, 50]. The Helsinki group has specialized in pediatric epilepsy surgery and published their results with respect to cognitive functions in a pediatric epilepsy surgery group some years ago [64].

### Historical dichotomy in the invasive diagnostic preoperative phase: Anglo-Saxon model versus French-Italian model

An example of the different strategies of analyzing a complex epilepsy surgery candidate is the way in which diagnostic electrode implantation is performed. Various types of electrodes exist: subdural grid and strip electrodes for covering the cortex versus depth electrodes for intracerebral positioning in deeper structures like the insular cortex, cingulate cortex, or hippocampus. The technique of recording electrical activity from the cortical pyramidal cells is called electrocorticography (ECoG) and can be done during (intraoperative) and after (extra-operative) the implantation procedure. This technique and its required electrodes have been introduced into clinical practice in the 1950s by the Montreal epilepsy group led by Penfield and Jasper [85]. Since these electrodes are implanted under the dura, the attenuating effect of low bone conductivity on the amplitude of the cortical potentials is not present, and for this reason, the spatial resolution of EcoG (1 cm) and temporal resolution of approximately 5 ms are clearly higher than those of the surface EEG [2, 36]. These electrodes have the advantage of a better delineation of the seizure onset zone and its propagation pathways, allowing more accurate resection of the epileptogenic zone and its margin. These electrodes have the disadvantage of high costs and long registration time combined with a potential morbidity risk. In many European countries, invasive seizure registration in complex patients is principally performed by implanting subdural electrodes frequently in combination with intrahippocampal depth electrodes. These electrodes have a dual function: extra-operative registration of seizure onset and spread versus extra-operative stimulation of eloquent cortex, usually for language mapping. This approach is appropriate for patients with a supposed epileptogenic focus on the cortical surface and a well-elaborated hypothesis on where this focus may probably be localized. In contrast to the above described implantation policy France (1960’s) and, three decades later, Italy, started with the SEEG. By this technique, intracerebral depth electrodes can be targeted precisely, by means of frame-based stereotactic or frame-less neuronavigational methods, to gray matter areas in the depth of the brain, e.g., the insula, with the purpose to detect the ictal onset zone and demarcate the epileptogenic zone. As MRI techniques become more advanced and consequently more subtle malformations of cortical development (MCD) being detected, the indications for use of SEEG, eventually in combination with surface electrodes, will increase. Nowadays, these conceptual differences in the preoperative workup still exist, but many epilepsy surgery working groups, including those in The Netherlands, the USA, and Germany also start using the SEEG technique next to the surface electrodes. There is a growing mondial tendency to combine these conceptually different invasive registration and stimulation techniques in the workup of epilepsy surgery candidates.

## Discussion and conclusion

**Fig. 7:**
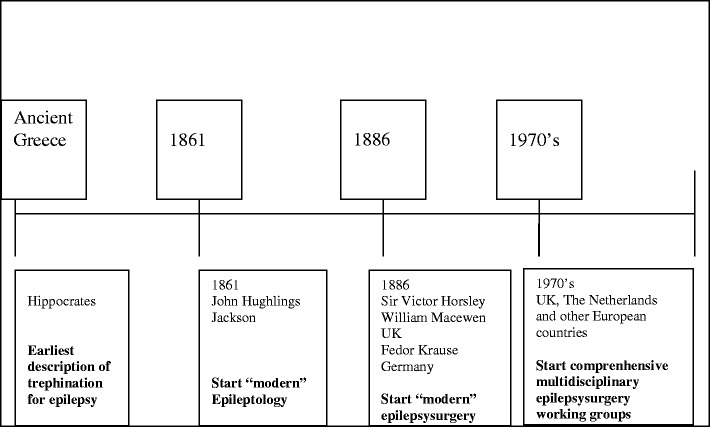
Schematic chronological trajectory “Development of Epileptology and Epilepsy surgery”

“Modern” epileptology and epilepsy surgery in Europe started (Fig. 7) in the second half of the nineteenth century synchronously in the UK with neurologist Hughlings Jackson in collaboration with neurosurgeon Sir Victor Horsley and in Germany with neurosurgeon Fedor Krause, inspired by the prior physiological work of neuropsychiatrist Eduard Hitzig. Hughlings Jackson was one of the first neurologists to suggest a causal relation between seizures and dysfunctional cortical neurons. At about the same time, Hitzig and Fritsch, in Berlin, demonstrated the cortical motor representations of the four extremities in the dog. The combination of both localization of functions by cortical electrostimulation in a clinical setting (Krause/Foerster) and experimental confirmation of this theory around the 1870s (Hitzig, Fritsch, Ferrier) has to be considered the forerunner traject of modern epilepsy surgery.

In the mid-1950s, the SEEG was introduced by Talairach and Bancaud in France, and in the mid-1990s, by Munari in Italy. Penfield and Jasper introduced electrocorticography with subdural electrodes in the 1950s in Montreal. This was followed by an introduction of these techniques in the 1960s and 1970s, in especially the Anglo-Saxon countries. This marked the start of a new era of invasive seizure registration. Nowadays, there is a growing tendency to use a combination of these two different implantation techniques tailored to the complexity of each patient. The introduction of the operation microscope and microsurgical instruments by well-known neurosurgeons as G. Yasargil and L. Malis (1919–2005), in addition to the advent of the bipolar coagulation forceps in the 1950s–1960s [71], led to an important step forward in microneurosurgery. In the years after the introduction of the operation microscope, important new resection techniques in the field of epilepsy surgery developed all over the world, including selective amygdalohippocampectomy [79, 114, 115] and functional hemispherotomy [91, 110, 111].

From the 1970s on, one saw an expansion in the number of epilepsy surgeries performed in Western European countries but also starting in Portugal and Spain at that time. In Greece, epilepsy surgery started two decades later at the end of the 1990s. With the advent of the MRI and its technological development, tiny (congenital) lesions could be visualized. After correlating these lesions to the ictal onset zone, more sophisticated microsurgical techniques, the use of neuronavigation and intraoperative 3-T MRI, lead to the performance of epilepsy surgical procedures with high success rates.

Today, a dedicated multidisciplinary epilepsy surgery working group is indispensable to establish an accurate radiological and neurophysiological diagnosis, to conceptualize the ictal onset zone, and to resect that specific part of the brain in order to obtain the highest chance for seizure freedom with the lowest risk for functional deficit. In almost all European centers for epilepsy surgery, nowadays, multidisciplinary working groups exist.

## Abbreviations

BC: Before Christ
SPECT: Single photon emission computed tomography
GABA: Gamma aminobutyric acid
DTI: Diffuse tensor imaging
ICP: Intracranial pressure
